# Health consumers’ use of mHealth applications in Ghana: a cross-sectional study

**DOI:** 10.1186/s12889-026-26996-z

**Published:** 2026-03-13

**Authors:** Dominic Dankwah Agyei, Dickson Dusi, Owusu Christopher Ntiamoah, Dake Daniella Nunya, Asiedu Charles Opoku Awuah, Christopher Ayisah

**Affiliations:** 1 University of Health and Allied Sciences, University Library, Ho, Ghana; 2 University of Health and Allied Sciences, School of Nursing and Midwifery, Ho, Ghana; 3 University of Health and Allied Sciences, Fred N. Binka School of Public Health, Ho, Ghana

**Keywords:** Smartphones, mHealth applications, Mobile health, Healthcare access, Digital literacy, Ghana

## Abstract

**Background:**

Mobile health (mHealth) applications are transforming healthcare delivery by enhancing access, reducing costs, and supporting remote health management. Despite growing global interest and adoption, awareness and utilisation of mHealth in Sub-Saharan Africa, including Ghana, remain unclear. This study aimed to scientifically assess the level of awareness and utilisation of mHealth applications among patients who use the Ho Teaching Hospital.

**Methods:**

A descriptive cross-sectional study design was conducted among 232 patients at the Ho Teaching Hospital. Systematic random sampling was utilised. Data were collected using a structured questionnaire and analysed using Stata version 17. Descriptive statistics, Chi-test, Fisher’s exact and logistic regression were employed to assess associations between sociodemographic variables and mHealth awareness, perception and usage. P-values of less than 0.05 were considered statistically significant at a 95% confidence level.

**Results:**

The majority of respondents were females 143 (61.6%), with a substantial proportion owning smartphones 162 (69.8%). Further, it was revealed that most 127(54.7%) of the respondents were unaware of mobile health (mHealth) apps. Over half of the respondents 123(53%) had a good perception of mHealth apps. A minority 79(34.1%) of the respondents had used mHealth apps, primarily for 56(53.3%) fitness tracking. The study found that awareness of mHealth applications was significantly associated with age, sex, education, occupation, and smartphone ownership. Perception of mHealth apps was predicted by sex, religion, education, smartphone ownership and awareness, while usage was significantly associated with various demographic factors, including age, marital status, ethnicity, education, occupation, and smartphone ownership.

**Conclusion:**

Despite high smartphone ownership, awareness and use of mHealth apps among patients at the Ho Teaching Hospital remain low. Targeted awareness campaigns and user education, especially among ethnic and religious groups, older, less-educated populations and smartphone owners, are essential to enhance adoption and optimise health outcomes through mHealth in Ghana.

**Supplementary Information:**

The online version contains supplementary material available at 10.1186/s12889-026-26996-z.

## Introduction

The use of information technology in healthcare settings has transformed healthcare delivery [1], and according to Bramo, it is influencing how health services are accessed and delivered [2]. It is moving healthcare from “industrial age medicine” to “information age healthcare”. Healthcare providers and consumers increasingly use information tools to dispense and receive services [3]. Information and Communication Technologies (ICTs) have bolstered access to health services, enhanced service quality, and reduced the cost of service delivery [4]. Over the years, one important innovation that has emanated from ICT enabled healthcare delivery is the advent of mobile Health (mHealth) applications, with Hoque and Sorwar arguing that mHealth applications probably provide some of the most prominent services with noticeable effects in clinical settings [5].

The rise of mobile Health applications (mHealth apps) has revolutionised the utilisation of mobile and wireless technologies in delivering healthcare services [6], and has altered the dynamics of user interactions with healthcare providers and the delivery of healthcare. mHealth encompasses medical applications operating on smartphones and tablets, sensors that monitor vital signs and health-related activities, and cloud-based computing systems for collecting health data [7]. Mobile health has also been defined as “medical and public health practice supported by mobile devices such as mobile phones, smartphones, tablets, patient monitoring devices, personal digital assistants, and other wireless devices” [8]. The aim of mHealth is to improve healthcare by making health information easily accessible to patients [9]. The focus on mHealth is growing as smartphone and tablet production increases, enabling easier access to the Internet and making them an integral part of the healthcare landscape. As of 2017, over 100,000 mobile applications were available [9].

Mobile health applications have significantly revolutionised and improved patient and healthcare providers’ relationships by offering better access and faster response times [6]. In recent times, the introduction of these applications in healthcare management has aided in overcoming geographical and organisational limitations, thereby enhancing the delivery of healthcare services [10]. In most cases, mHealth is utilised to disseminate healthcare information to the general population, gather health-related data, oversee patients from a remote location, retrieve health records, provide medical diagnoses, and aid in disease prevention and control [11].

The ownership and use of smartphones and other mobile devices have increased significantly over the last decade. In 2018, 36% of the global population owned smartphones, up from 10% in 2011 [12]. Since 2015, the ownership of smartphones among adults has increased to 71%, while among young adults under 34 years old, it is estimated to be around 90% [9]. According to data from the Global System for Mobile Communications Association (GSMA), mobile phone penetration in Sub-Saharan Africa (SSA) reached 51%, with 495 million individuals, making up 46% of the population, having mobile service subscriptions as of the end of 2020 [8, 13]. The current count of Internet users in Sub-Saharan Africa stands at 303 million, constituting 28% of the population, and is expected to rise to 474 million by 2025 [8, 13].

Again, more Sub-Saharan African nations are adopting mHealth to increase accessibility to high-quality, egalitarian healthcare, particularly for underprivileged and vulnerable people [8]. Apps, online media, radio, landlines, television, telemedicine, Short Message Service (SMS) text, wearables, and other text-messaging devices are just a few of the technology tools that are used in mHealth approaches [8]. In 2018, approximately half of mobile phone users had at least one mobile health application installed [14].

In the healthcare industry, emerging technologies are used for medical training and practice. These technologies take the form of educational apps designed for medical professionals. These apps include medical terminologies, drug-referencing, and clinical decision-support tools [15]. mHealth applications focused on health and wellness help encourage healthy habits, especially among young people and students. These wellness applications often include step trackers, calorie trackers, tools for managing weight loss, and workout plans [16]. Medical applications can be grouped into medical education and instructional tools, health and wellness applications for patients and the public, and telemedicine and telehealth applications [17].

Ghana and the other nations in the SSA region deal with the problem of a combined burden of non-communicable and communicable diseases [18]. In addition, these nations have inadequate healthcare systems [19, 20]. The problem is made worse by elements like poor road systems, lengthy travel times to medical institutions, a lack of funding, a lack of health education, and underqualified medical personnel [21, 22]. The advent of mHealth technologies has had a significant impact on addressing some of these healthcare challenges in Sub-Saharan Africa (SSA) [8]. For instance, mHealth applications have been used to assist in disease diagnosis and treatment, manage chronic conditions, and provide health education [8]. These technologies help reduce the need to travel to medical facilities by enabling remote consultations and monitoring, thus overcoming geographical barriers [22]. Furthermore, mHealth initiatives have improved the training and support of healthcare workers, thereby enhancing the quality of care provided [22]. By addressing funding and resource constraints, mHealth has also facilitated more efficient use of available resources, contributing to improved health outcomes in the region [8].

Poor public adoption of mHealth persists despite the many documented advantages it offers. Socioeconomic issues have been recognised as a barrier to the widespread use of mHealth, and its adoption varies across demographics [17]. Additionally, there is still doubt about whether mHealth can lead to better patient outcomes and habits in this part of the world [23]. The aim of this study was to scientifically assess the level of awareness and utilisation of mHealth applications among patients who use the Ho Teaching Hospital.

## Methods

### Study design and setting

The quantitative cross-sectional design used in this study was selected for its ability to estimate prevalence. The method is suitable for a population-based study because it is affordable, enables rapid variable measurement, and doesn’t require follow-up [24, 25]. Compared to other study designs, cross-sectional designs are less expensive and provide an objective means of measuring phenomena over a brief period of time without requiring participant follow-up [25], even though cross-sectional studies are vulnerable to nonresponse and recall bias [24, 26].

The study was carried out at the Ho Teaching Hospital, the fifth public teaching hospital in Ghana, located in the Volta Region. The hospital occupies a strategic position, enabling it to provide specialised healthcare services to residents of the Volta Region and neighbouring countries. It serves clients from Togo, Benin, and Nigeria and aims to position itself as a leading medical tourism centre through the delivery of innovative tertiary healthcare, medical education, and research [27].

### Study population

The study population consisted of outpatients who sought healthcare at the Ho Teaching Hospital. The study included patients aged 18 years and above who sought care at the teaching hospital and provided informed consent. Individuals who refused to participate, those who were mentally unstable, or in critical conditions were excluded.

### Sampling method

Data collection took place on a single clinic day in July 2024. The sampling frame consisted of all eligible patients who attended the Outpatient Department (OPD) on that clinic day. The facility’s attendance register indicated an average daily OPD attendance of 489 clients over the preceding month. Because sampling was restricted to one clinic day and recruitment occurred only among patients present that day, this value was used as an estimate of the finite accessible population (*N* = 489). To determine the sample size for this study, the Yamane formula was used, with n representing the required sample size, N the estimated accessible population, and e is the margin of error (0.05).

$$n=\frac{N}{1+N\left(e\right)^{2}}$$

Substituting into the formula:

$$n=\frac{489}{1+489\left(0.05\right)^{2}}=220$$

To account for a 5% non-response rate, the sample size was adjusted as follows:

$$n=\frac{220}{0.95}=232$$

Therefore, the minimum required sample size was 232 respondents.

### Sampling procedure

Respondents were selected using a systematic random sampling approach. Eligible patients attending the OPD were identified from the daily attendance register and arranged in the order of their arrival.

The sampling interval (k) was calculated by dividing the average daily attendance (489) by the required sample size (232), yielding an interval of 2. Therefore, every second eligible patient was selected for participation. A random starting point was chosen among the first two eligible attendees to ensure unbiased selection. If a selected participant declined participation or did not meet eligibility criteria, the next eligible patient on the register was approached to maintain the sampling sequence. A total of 244 eligible patients were approached, of whom 232 consented and completed the survey, yielding a response rate of 95%. This systematic sampling ensured a more representative selection of respondents across the study population.

### Data collection tool and procedure

Data collection was conducted using Google Forms with a structured questionnaire developed solely for this study (sample questionnaire attached as a supplementary file). The questionnaire included three sections: socio-demographic characteristics of the outpatients, level of awareness of mHealth applications among patients, and attitudes and perceptions regarding the use of mHealth applications and their effectiveness. The instrument was pre-tested among 30 respondents in two private hospitals in the Ho Municipality and excluded from the final analysis. Revisions based on feedback were made to ensure all items were clear, relevant, and easy to understand. Reliability was acceptable with Cronbach’s alpha of 0.733. We minimised potential bias by ensuring anonymity and confidentiality throughout data collection and by training data collectors to encourage truthful reporting. Four trained data collectors administered the survey within the OPD on the same clinic day. For respondents with low literacy, the questionnaire had to be administered orally by the interviewer in an enclosed room to ensure privacy and confidentiality. After completion of this process, the questionnaires were carefully observed and coded for data analysis. Completion time ranged from 15 to 30 min.

### Study variables

The main outcome variables were the use of mHealth applications, awareness, and perceptions of mHealth applications. Usage of mHealth applications was assessed using the following variable: “ever used any mHealth App”. Moreover, awareness was assessed using the following variable: “Are you aware of what we call Mobile Health (mHealth)?”. Perception was measured using 9 variables (see supplementary file). Correct responses were summed and categorised as good (≥ 50% of total score) or poor (< 50%) perception based on the median split of the composite scores [28, 29]. The independent variables consisted of respondents’ individual background characteristics, including age, sex, religion, ethnicity, educational level, and occupational status.

### Data analysis

The collected data were exported into STATA version 17.0 for cleaning and analysis. Data cleaning involved removing duplicates and correcting errors to ensure accuracy. Descriptive statistics, including means or medians, frequencies, and proportions, were used to summarise the socio-demographic characteristics and outcome variables. Inferential statistics, such as Chi-square tests, Fisher’s Exact Test, and logistic regression, were used to assess associations between demographic factors and outcome variables. To ensure no serious multicollinearity, Variance Inflation Factors (VIFs) and Tolerance values were calculated separately for the sociodemographic/Independent variable. All variables included in the final models had VIFs below 2.0, indicating acceptable variation among independent variables. The Hosmer-Lemeshow test for usage of mHealth applications (*p* = 0.13), awareness (*p* = 0.27), and Perception (*p* = 0.48) indicated no significant deviation between observed and predicted values. Statistical significance was determined at *p* < 0.05, and a 95% confidence interval was used to estimate the precision of the results.

### Ethics approval and consent to participate

The study received ethical clearance from the University of Health and Allied Sciences (UHAS) Research Ethics Committee (REC) (UHAS-REC A.11H [18]23–24) and approval from the Ho Teaching Hospital. Before data collection, written informed consent was obtained from all respondents (above 18 years) after they had been fully briefed on the study’s purpose. During the consent process, all eligible respondents were informed of their freedom to leave the study at any time without affecting the health care they received. After the study procedures had been explained, informed consent was obtained from all respondents.

On the other hand, for respondents with low literacy, a witness was present, and verbal consent was duly documented. Confidentiality was ensured by assigning unique identification codes, limiting data access to only the research team, and securely storing all records with passcodes. For respondents with low literacy who required interviewer administered questionnaires, interviews were conducted in private settings, and trained interviewers adhered strictly to confidentiality protocols. Respondents were informed that their responses would be kept confidential and would not affect their care. No identifying information was included in the study findings. Respondents were informed that there would be no direct personal benefit, but that the findings would inform relevant policy recommendations.

## Results

This study saw more females 143(61.6%) participating in the survey. Again, it was realised that most of the respondents were Christians 202(87.0%). It was also found that most respondents had attained tertiary education, with most of them reporting as gainfully employed 137(59.1%). Majority 162(69.8%) of the respondents reported owning a smartphone as shown in Table 1.

**Table 1:** Demographic characteristics of respondents

Variable	Frequency (232)	Percentage (%)
Age		
18–24	24	10.3
25–34	65	28.0
35–44	64	27.6
45 and above	79	34.1
Sex		
Male	89	38.4
Female	143	61.6
Marital status		
Single	82	35.3
Married / Cohabiting	128	55.3
Divorced / Separated	11	4.7
Widowed	11	4.7
Religion		
Christian	202	87.0
Muslim	25	10.8
African Traditional Religion	5	2.2
Ethnic background		
Ewe	141	60.8
Akan	38	16.4
Hausa	25	10.8
Ga/Dangme	12	5.2
Fante	11	4.6
Others	5	2.2
Educational level		
Basic	55	23.7
Secondary	62	26.7
Tertiary	87	37.5
No formal education	28	12.1
Occupation		
Employed / Self-Employed	137	59.1
Unemployed	36	15.5
Retired	32	13.8
Student	27	11.6
Ownership of smartphone(s)		
Yes	162	69.8
No	70	30.2

### Knowledge of mHealth apps

**Fig. 1:**
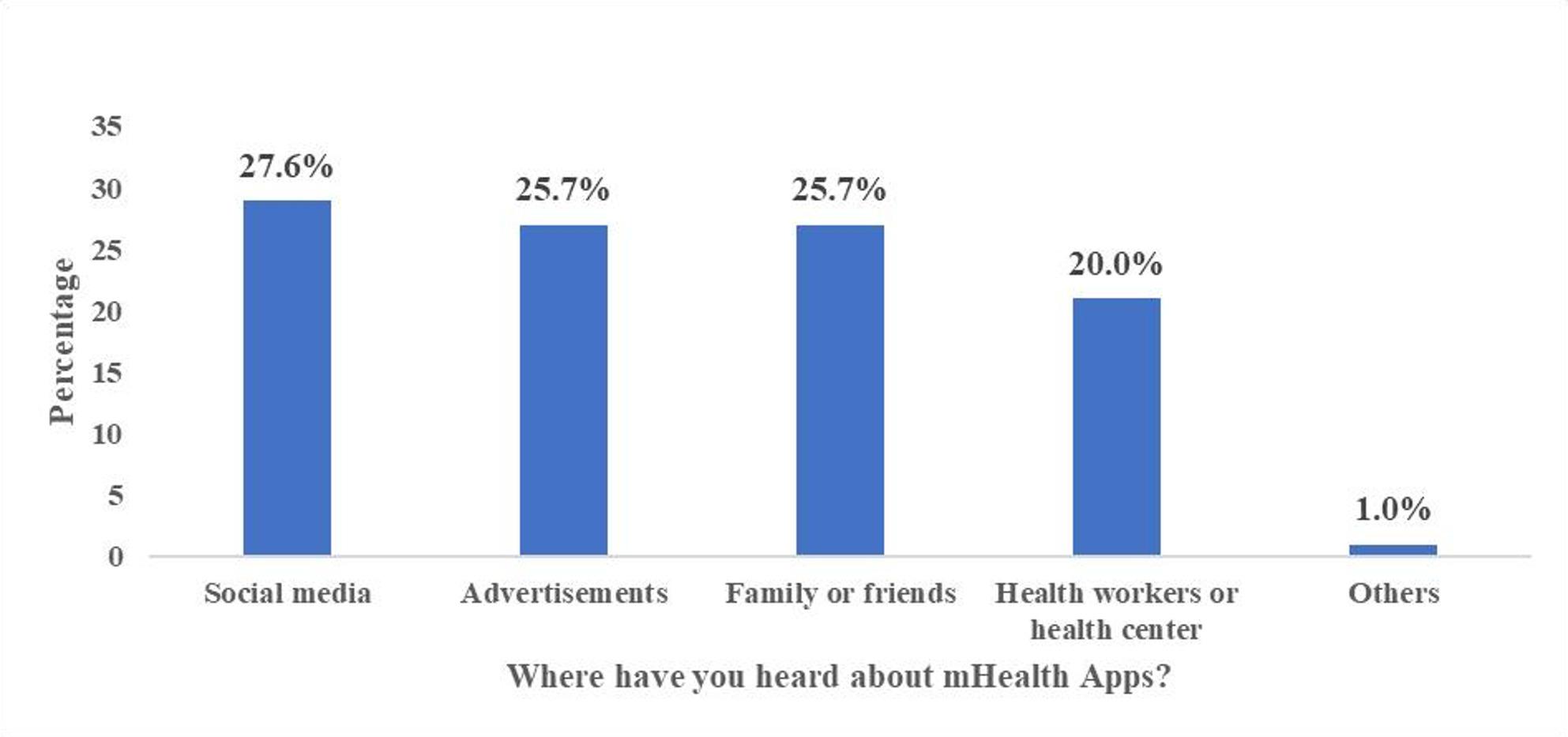
Source of knowledge on mHealth apps

Among those who had knowledge about the apps, it was found that social media contributed 27.6% to their source of knowledge (Fig. 1**)**.

The study further indicated that most of the respondents 56(53.3%) were familiar with fitness apps, followed by vital signs tracker apps 46(43.8%), my calendar (period tracker) 43(41.0%), diet and nutrition apps 17(16.2%), and then knowing medical education apps 11(10.5%) as shown in Table 2.

**Table 2:** Respondents’ knowledge of mHealth apps

Parameters	Frequency (105)	Percentage (%)
Fitness Apps	56	53.3
Vital signs tracker apps	46	43.8
My calendar (period tracker)	43	41.0
Medical education apps	11	10.5
Diet and nutrition apps	17	16.2

### Awareness of mHealth application

**Fig. 2:**
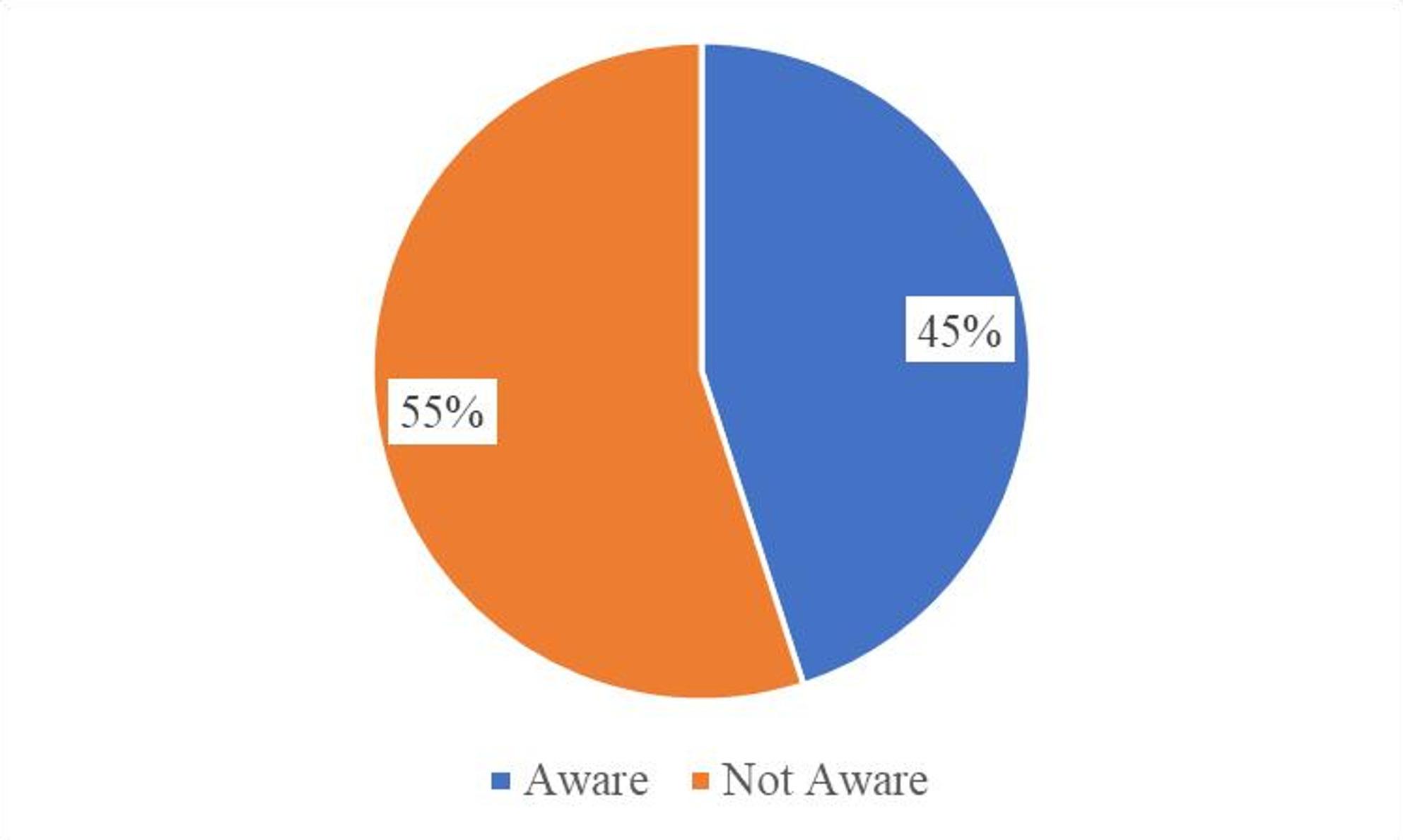
Awareness of mHealth application

As indicated in Fig. 2, this study revealed that more than half of the respondents, 127(54.7%), were not aware of mHealth applications, while 105 (45.3%) of the respondents were aware.

### Utilisation of mHealth applications

The study found that only 79 (34.1%) of the respondents reported using at least one mHealth app. Regarding the specific types of apps used, 42 (53.2%) used fitness apps, while 4 (5.1%) used medical education apps. Among those who used the apps, 22 (27.8%) used them a few times a week, while 16 (20.3%) used them occasionally. Also, it was found out that 37 (46.8%) used them because they help track their health, and 6 (7.6%) used them for their user-friendliness. A significant number of respondents who used the apps 78 (98.7%), indicated their willingness to recommend them to their friends or families (Table 3).

**Table 3:** Respondents’ use of mHealth apps

Parameters	Frequency (232)	Percentage (%)
Ever used any mHealth App		
No	153	65.9
Yes	79	34.1
Usage of mHealth apps	*n* = 79	
Fitness Apps	42	53.2
Vital signs tracker apps	24	30.4
My calendar (period tracker)	27	34.2
Medical education apps	4	5.1
Diet and nutrition apps	13	16.5
Frequency of use		
Daily	20	25.3
A few times a week	22	27.8
A few times a month	21	26.6
I barely use them	16	20.3
Reasons for using mHealth apps		
Helps me track my health	37	46.8
It motivates me	13	16.5
It provides useful reminders	23	29.1
It is fun or simple to use	6	7.6
Willing to recommend mHealth Apps	78	98.7

### Association between factors influencing awareness of mHealth apps

Table 4 shows that age (χ² = 22.989, p = < 0.001), marital status (*p* = 0.001), ethnic background (p = < 0.001), educational level (p = < 0.001), Occupational status (χ² =15.137, *p* = 0.002) and smartphone ownership (χ² = 50.302, *p* < 0.001) were statistically significant with perception of mHealth application.

**Table 4:** Chi-square analysis results on awareness of mHealth apps associated with respondents’ sociodemographic characteristics

Covariate	Awareness of mHealth Application		X^2^(p-value)	Fisher’s Exact Test Stat.
	Not Aware 127 (54.7%)	Aware 105(45.3%)		
Age				
18–24	13(54.2)	11(45.8)	**22.989(** **< 0.001)**	
25–34	27(41.5)	38(58.5)		
35–44	27(42.2)	37(57.8)		
45 and above	60(75.9)	19(24.1)		
Sex				
Male	43(48.3)	46(51.7)	2.407(0.121)	
Female	84(58.7)	59(41.3)		
Marital status				
Single	30(36.6)	52(63.4)		**0.001**
Married/Cohabiting	83(64.8)	45(35.2)		
Divorced/Separated	7(63.6)	4(36.4)		
Widowed	7(63.6)	4(36.4)		
Religion				
Christian	110(54.5)	92(45.5)		1.000
Muslim	14(56.0)	11(44.0)		
African Traditional Religion	3(60.0)	2(40.0)		
Ethnic background				
Ewe	58(41.1)	83(58.9)		**< 0.001**
Akan	23(60.5)	15(39.5)		
Hausa	21(84.0)	4(16.0)		
Ga/Dangme	7(58.3)	5(41.7)		
Fante	0(0)	11(100)		
Others	3(60.0)	2(40.0)		
Educational level				
No formal education	25(89.3)	3(10.7)		**< 0.001**
Basic	50(90.9)	5(9.1)		
Secondary	36(58.1)	26(41.9)		
Tertiary	16(18.4)	71(81.6)		
Occupational status				
Employed/Self-Employed	73(53.3)	64(46.7)	**15.137(0.002)**	
Unemployed	26(72.2)	10(27.8)		
Retired	21(65.6)	11(34.4)		
Student	7(25.9)	20(74.1)		
Ownership of smartphone				
Yes	64(39.5)	98(60.5)	**50.302(< 0.001)**	
No	63(90.0)	7(10.0)		

The results in Table 5 indicate that all variance inflation factor (VIF) values for the sociodemographic variables are well below the generally accepted cutoff of 5, ranging from 1.08 (occupational status) to 1.96 (awareness of mHealth apps). A low level of multicollinearity among the independent variables is further indicated by a mean VIF of 1.46. Also, the tolerance values or the 1/VIF range from 0.610 to 0.927, all of which are above the minimum threshold of 0.20. This indicates that each independent variable accounts for an adequate proportion of its own variance and that none are highly associated with others.

**Table 5:** Multicollinearity diagnostics (VIF) for sociodemographic variables

Independent Variable	VIF	Tolerance (1/VIF)
Age	1.71	0.69
Sex	1.17	0.85
Marital Status	1.67	0.69
Religion	1.14	0.88
Ethnic background	1.20	0.83
Educational level	1.21	0.82
Occupational status	1.08	0.93
Ownership of smartphone	1.58	0.63
Awareness of mHealth Apps	1.96	0.61
Perception of mHealth Apps	1.87	0.63
Mean VIF	1.46	

### Factors associated with awareness of mHealth apps

Table 6 shows that respondents aged 25–34 (AOR = 16.08, 95% CI: 3.13–82.74, *p* = 0.001) and 35–44 (AOR = 19.59, 95% CI: 2.91–31.93, *p* = 0.002) were significantly more likely to be aware of mHealth applications compared to those aged 18–24. Females were less likely to be aware than males (AOR = 0.32, 95% CI: 0.12–0.81, *p* = 0.017). Respondents with basic education had lower odds of awareness compared to those with no formal education (AOR = 0.12, 95% CI: 0.02–0.88, *p* = 0.037). Students were significantly more likely to be aware than employed individuals (AOR = 11.96, 95% CI: 2.08–68.67, *p* = 0.005). Respondents without a smartphone were less likely to be aware of mHealth applications compared to smartphone owners (AOR = 0.25, 95% CI: 0.07–0.86, *p* = 0.028).

**Table 6:** Factors associated with awareness of mHealth apps among respondents

Covariate	cOR (95% CI)	*p*-Value	AOR (95% CI	*p*-Value
Age				
18–24	1		1	
25–34	1.66(0.65–4.27)	0.29	16.08(3.12–82.74)	0.001***
35–44	1.62(0.63–4.16)	0.317	19.59(2.91–31.93)	0.002***
45 and above	0.37(0.14–0.97)	0.044**	4.01(0.49–32.14)	0.191
Sex				
Male	1		1	
Female	0.66(0.39–1.12)	0.122	0.32(0.12–0.81)	0.017**
Marital status				
Single	1		1	
Married/Cohabiting	0.31(0.18–0.56)	0.000***	0.72(0.24–2.17)	0.555
Divorced/Separated	0.33(0.09–1.22)	0.096*	2.33(0.23–23.83)	0.475
Widowed	0.33(0.9–1.22)	0.096*	1.31(0.58–29.57)	0.865
Religion				
Christian	1		1	
Muslim	0.94(0.41–2.17)	0.884	0.37(0.09–1.35)	0.131
African Traditional Religion	0.79(0.13–4.87)	0.806	2.99(0.20-13.94)	0.424
Ethnic background				
Ewe	1		1	
Akan	2.18(1.06–4.51)	0.035***	0.74(0.26–2.15)	0.584
Hausa	1.39(0.59–3.28)	0.457	1.21(0.38–3.79)	0.748
Ga/Dangme	2.47(0.75–8.19)	0.139	1.87(0.29–12.18)	0.515
Others	7.06(0.77–64.87)	0.084*	0.98(0.75–12.71)	0.986
Educational level				
No formal education	1		1	
Basic	0.83(0.18–3.77)	0.813	0.12(0.02–0.88)	0.037**
Secondary	6.02(1.64–22.07)	0.007**	0.61(0.09–3.92)	0.602
Tertiary	36.98(9.93-137.69)	0.000***	4.32(0.74–25.33)	0.105
Occupational status				
Employed/Self-Employed	1			
Unemployed	0.44(0.19–0.98)	0.044**	0.43(0.13–1.39)	0.159
Retired	0.59(0.27–1.33)	0.209	1.69(0.38–7.63)	0.495
Student	3.26(1.29–8.21)	0.012**	11.96(2.08–68.67)	0.005***
Ownership of smartphone				
Yes	1		1	
No	0.07(0.03–0.17)	0.000***	0.25(0.07–0.86)	0.028**

*** *p* < 0.01, ** *p* < 0.05, * *p* < 0.1

### Perceptions regarding mHealth apps

Another objective of the study was to determine patients’ perceptions of mHealth apps. It was found that more than half of the respondents 141 (60.8%) were willing to try or use mHealth apps to manage their personal health. It was also revealed that 112 (48.3%) believed that such apps help save time, including the time spent going to health facilities for simple information, even though only 66 (28.4%) believed that these apps can help them access more accurate and reliable health information, as shown in Table 7.

**Table 7:** Respondents’ perceptions regarding mHealth apps

Parameters	Frequency (232)	Percentage (%)
Willingness to try or use mHealth apps	141	60.8
Time saving	112	48.3
Efficient management of healthcare needs	110	47.4
Increased ability to understand my own health conditions	105	45.3
Cost effectiveness	105	45.3
Willingness to use mHealth apps without endorsement from trusted authorities	99	42.7
Using these apps is safe and secure	98	42.2
Trust to share personal or contact details	75	32.3
Access to more reliable and accurate information	66	28.4

### Perception levels among respondents

**figure. 3:**
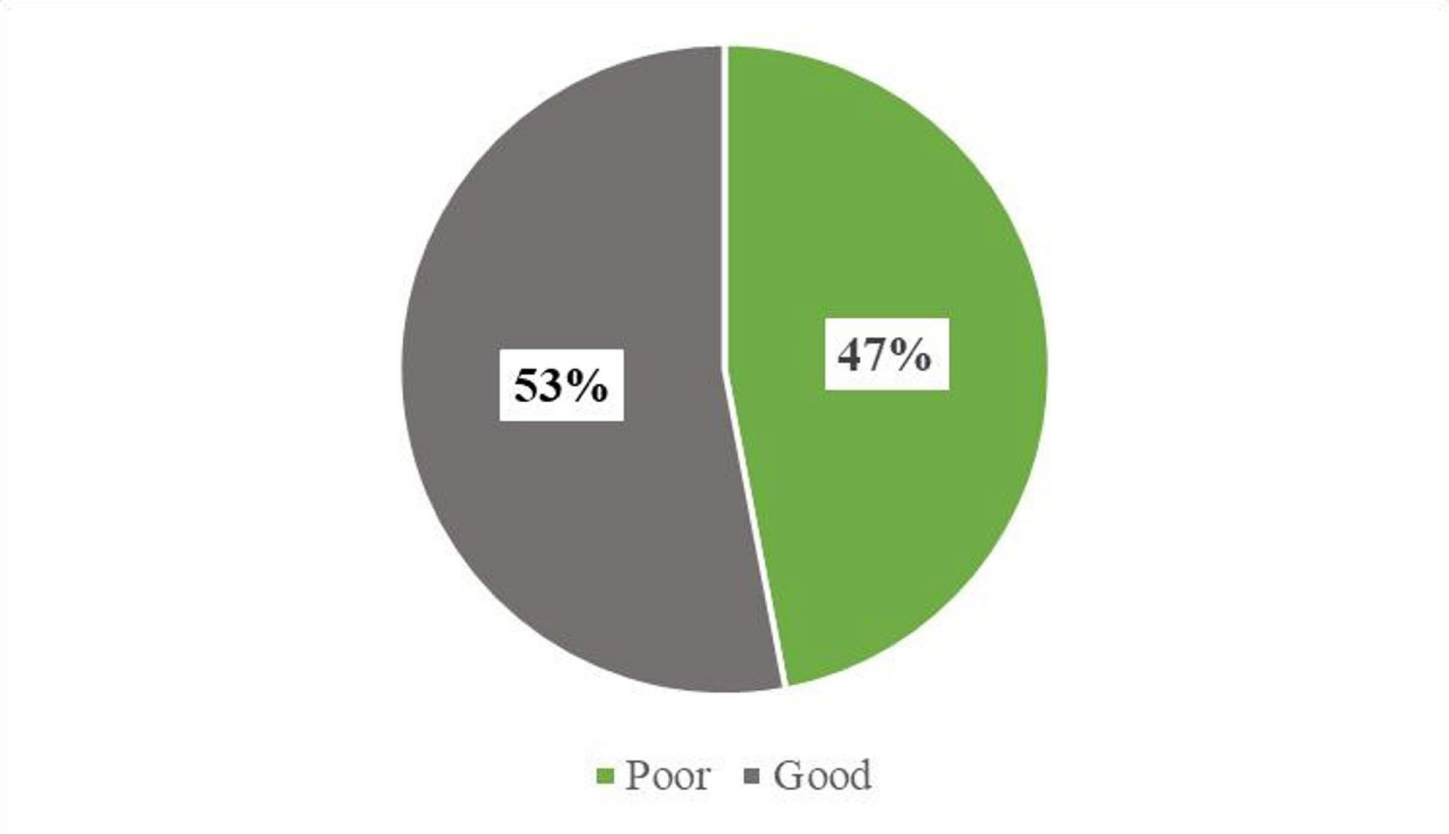
Respondents’ perception levels regarding mhealth usage

Further, this study explored respondents’ perceptions of mHealth applications. It was found that just over half of the respondents 123(53%) had a good perception of using mHealth, while 109(47%) had a poor perception. Figure 3 demonstrates how respondents perceive mHealth applications.

### Association between factors influencing perception of mhealth apps

Table 8 shows that age (χ² = 22.353, p = < 0.001), sex (χ² = 12.018, *p* = 0.001), marital status (χ² = 8.738, *p* = 0.033), religion (*p* = 0.002), ethnic background (p = < 0.001), educational level (p = < 0.001), smartphone ownership (χ² = 51.795, *p* < 0.001) and awareness (χ² = 87.191, p = < 0.001) were statistically significant with perception of mHealth application.

**Table 8:** Chi-square analysis results on perception of mHealth apps associated with respondents’ sociodemographic characteristics

Covariate	Perception of mHealth Application		X^2^(*p*-value)	Fisher’s Exact Test Stat.
	Poor 123 (53%)	Good 109(47%)		
Age				
18–24	10(41.7)	14(58.3)	**22.353(< 0.001)**	
25–34	23(35.4)	42(64.6)		
35–44	22(34.4)	42(65.6)		
45 and above	54(68.4)	25(31.7)		
Sex				
Male	29(32.6)	60(67.4)	**12.018(0.001)**	
Female	80(55.9)	63(44.1)		
Marital status				
Single	28(34.2)	54(65.9)	**8.738(0.033)**	
Married/Cohabiting	70(54.7)	58(45.3)		
Divorced/Separated	5(45.5)	6(54.6)		
Widowed	6(54.6)	5(45.5)		
Religion				
Christian	102(50.5)	100(49.5)		**0.002**
Muslim	4(16.0)	21(84.0)		
African-Traditional Religion	3(60.0)	2(40.0)		
Ethnic background				
Ewe	83(58.9)	58(41.1)		**< 0.001**
Akan	15(39.5)	23(60.5)		
Hausa	4(16.0)	21(84.0)		
Ga/Dangme	5(41.7)	7(58.3)		
Fante	0(0.0)	11(100)		
Others	2(40.0)	3(60.0)		
Educational level				
No formal education	28(100)	0(0)		**< 0.001**
Basic	37(67.3)	18(32.7)		
Secondary	28(45.2)	34(54.8)		
Tertiary	16(18.4)	71(81.6)		
Occupational status				
Employed/Self-Employed	60(43.8)	77(56.2)	6.413(0.093)	
Unemployed	21(58.3)	15(41.7)		
Retired	19(59.4)	13(40.6)		
Student	9(33.3)	18(66.7)		
Ownership of smartphone				
Yes	51(31.5)	111(68.5)	**51.795(< 0.001)**	
No	58(82.9)	12(17.1)		
Awareness of mHealth App				
Not Aware	95(74.8)	32(25.2)	**87.191(< 0.001)**	
Aware	14(13.3)	91(86.7)		

### Factors influencing perception of mHealth apps

Table 9 shows that females were less likely than males to have a positive perception (AOR = 0.42, 95% CI: 0.19–0.87, *p* = 0.021). Muslims were more likely than Christians to have a positive perception (AOR = 4.83, 95% CI: 1.28–18.22, *p* = 0.020). Respondents with basic (AOR = 0.21, 95% CI: 0.07–0.56, *p* = 0.002) and secondary education (AOR = 0.16, 95% CI: 0.06–0.43, *p* < 0.001) were less likely to have positive perceptions compared to those with tertiary education. Respondents without smartphones were less likely to have a positive perception than smartphone owners (AOR = 0.27, 95% CI: 0.09–0.76, *p* = 0.013). Respondents who were aware of mHealth were approximately 19 times more likely to have a positive perception about it (AOR = 18.63, 95% CI: 6.61–52.48, *p* < 0.001).

**Table 9:** Factors associated with perception of mHealth apps among respondents

Covariate	cOR (95% CI)	*p*-Value	AOR (95% CI	*p*-Value
Age				
18–24	1		1	
25–34	1.3(0.50–3.39)	0.0587	1.45(0.45–4.68)	0.532
35–44	1.36(0.52–3.57)	0.527	1.07(0.25–4.64)	0.928
45 and above	0.33(0.13–0.85)	0.021**	0.71(0.16–3.13)	0.652
Sex				
Male	1		1	
Female	0.38(0.22–0.66)	0.001**	0.42(0.19–0.87)	0.021**
Marital status				
Single	1		1	
Married/Cohabiting	0.43(0.24–0.76)	0.004**	0.91(0.33–2.46)	0.846
Divorced/Separated	0.62(0.17–2.22)	0.465	1.54(0.20-11.65)	0.679
Widowed	0.43(0.12–1.54)	0.196	1.81(0.25–13.31)	0.561
Religion				
Christian	1		1	
Muslim	5.4(1.77–16.16)	0.003**	4.83(1.28–18.22)	0.020***
African Traditional Religion	0.68(0.11–4.16)	0.676	0.97(0.12–8.13)	0.976
Ethnic background				
Ewe	1		1	
Akan	2.19(1.6–4.56)	0.035	1.17(0.47–2.93)	0.732
Hausa	7.51(2.45–23.04)	< 0.001***	2.26 (0.58–8.78),	0.167
Ga/Dangme	2.0(0.61–6.62)	5	0.89(0.21–3.82)	0.884
Others	2.15(0.35–13.25)	0.411	0.38(0.05–2.92)	0.353
Educational level				
No formal education	1		1	
Basic	0.11(0.05–0.24)	< 0.001***	0.21(0.07–0.56)	0.002***
Secondary	0.27(0.13–0.57)	0.001**	0.16(0.06–0.43)	< 0.001***
Occupational status				
Employed/Self-Employed	1		1	
Unemployed	0.56(0.26–1.17)	0.122	1.64(0.48–5.58)	0.430
Retired	0.53(0.24–1.17)	0.115	0.83(0.23–2.94)	0.774
Student	1.56(0.65–3.71)	0.317	1.68(0.43–6.50)	0.454
Ownership of smartphone				
Yes	1		1	
No	0.09(0.05–0.19)	< 0.001***	0.27(0.09–0.76)	0.013**
Awareness of mHealth App				
Not aware	1		1	
Aware	19.29(9.67–38.49)	< 0.001***	18.63(6.61–52.48)	< 0.001***

*** *p* < 0.01, ** *p* < 0.05, * *p* < 0.1

### Barriers in mHealth Utilisation

**Fig. 4:**
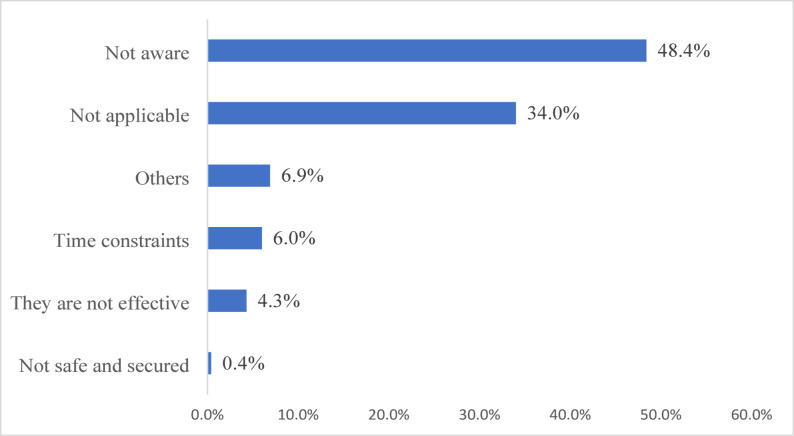
Respondents’ reasons for not using mHealth apps

Further, the study sought to identify the reasons some respondents never used any mHealth apps. In light of this, it was observed that the majority of respondents 112 (48.2%) were not aware of the existence of such apps. However, only a small percentage cited specific concerns, such as mHealth not being effective 10 (4.3%), as shown in Fig. 4.

### Association between factors influencing usage of mHealth apps

To understand which factors were associated with the adoption and use of mHealth apps, this study conducted chi-square tests comparing respondents’ sociodemographic characteristics with their app use. It was revealed that respondents’ age (χ² = 18.441, p = < 0.001), marital status (*p* = 0.001), ethnic background (p = < 0.001), educational status (p = < 0.001), occupational status (*p* = 0.004), ownership of smartphones (χ² = p = < 0.001), awareness (p = < 0.001) and perception (χ² =69.891, p = < 0.001) were all statistically linked to the use of mHealth apps, with p-values less than 0.05 as shown in Table 10.

**Table 10:** Chi-square analysis results on the use of mHealth apps associated with respondents’ sociodemographic characteristics

Variable	Use of mHealth Applications			X^2^(*p*-value)	
	Total (232)	Users 79(34.1%)	Non-users 153(65.9%)		Fisher’s Exact Test Stat.
Age					
18–24	24	7(29.2)	17(70.8)	**18.441(< 0.001)**	
25–34	65	33(50.8)	32(49.2)		
35–44	64	25(39.1)	39(60.9)		
45 and above	79	14(17.7)	65(82.3)		
Sex					
Male	89	29(32.6)	60(67.4)	0.138(0.710)	
Female	143	50(35.0)	93(65.0)		
Marital status					
Single	82	41(50.0)	41(50.0)		**0.001**
Married / Cohabiting	128	30(23.4)	98(76.6)		
Divorced / Separated	11	4(36.4)	7(63.6)		
Widowed	11	4(36.4)	7(63.6)		
Religion					
Christian	202	66(32.7)	136(67.3)		0.520
Muslim	25	11(44.0)	14(56.0)		
African Traditional Religion	5	2(40.0)	3(60.0)		
Ethnic background					
Ewe	141	33(23.4)	108(76.6)		**< 0.001**
Akan	38	16(42.1)	22(57.9)		
Hausa	25	11(44.0)	14(56.0)		
Ga/Dangme	12	5(41.7)	7(58.3)		
Fante	11	11(100.0)	0(0.0)		
Others	5	3(60.0)	2(40.0)		
Educational level					
Basic	55	1(1.8)	54(98.2)		**< 0.001**
Secondary	62	18(29.0)	44(71.0)		
Tertiary	87	59(67.8)	28(32.2)		
No formal education	28	1(3.6)	27(96.4)		
Occupational status					
Employed / Self-Employed	137	46(33.6)	91(66.4)	**13.490(0.004)**	
Unemployed	36	9(25.0)	27(75.0)		
Retired	32	7(21.9)	25(78.1)		
Student	27	17(63.0)	10(37.0)		
Ownership of smartphone					
Yes	162	75(46.3)	87(53.7)	**35.847(< 0.001)**	
No	70	4(5.7)	66(94.3)		
Awareness of mHealth App					
Not aware	127	0(0)	127(100)		**< 0.001**
Aware	105	79(75.2)	26(24.8)		
Perception of mHealth apps					
Poor	109	7(6.4)	102(93.6)	**69.891(< 0.001)**	
Good	123	72(58.5)	51(41.5)		

### Factors influencing usage of mHealth Apps

To examine factors associated with the use of mHealth apps, bivariate and multivariate logistic models were estimated. After controlling for potential confounders, age, educational level and occupation remained significant factors. Respondents aged 25–34 were over 14 times more likely to use mHealth apps compared to those aged 18–24 (AOR = 14.14, 95% CI: 2.21–90.32, *p* = 0.005). Individuals with tertiary education were about 18 times more likely to use mHealth apps than those without formal education (AOR = 17.76, 95% CI: 1.32–23.52, *p* = 0.030). Also, students were approximately 8 times more likely to use mHealth apps (AOR = 7.76, 95% CI: 1.10-54.59, *p* = 0.040) as shown in Table 11.

**Table 11:** Multivariate logistic regression of factors associated with the use of mHealth apps

Covariate	cOR (95% CI)	*p*-Value	AOR (95% CI	*p*-Value
Age				
18–24	1		1	
25–34	2.50 (0.92–6.85)	0.074*	14.14 (2.21–90.32)	0.005***
35–44	1.56 (0.57–4.29)	0.392	5.63 (0.75–42.18)	0.093*
45 and above	0.23 (0.18–1.49)	0.228	5.69 (0.49–65.45)	0.163
Sex				
Male	1		1	
Female	1.11 (0.63–1.95)	0.710	1.07 (0.42–2.70)	0.887
Marital status				
Single	1		1	
Married/Cohabiting	0.31 (0.17–0.56)	< 0.001***	0.42 (0.14–1.32)	0.138
Divorced/Separated	0.57 (0.16–2.10)	0.400	1.49 (0.09–24.19)	0.781
Widowed	0.57 (0.16–2.10)	0.400	2.0 (1.62–9.68)	0.990
Religion				
Christian	1		1	
Muslim	1.62 (0.69–3.76)	0.262	3.25 (0.83–12.73)	0.091*
African Traditional Religion	1.37 (0.22–8.42)	0.731	8.11 (4.63–12.81)	0.986
Ethnic background				
Ewe	1		1	
Akan	2.38 (1.12–5.05)	0.024**	1.19 (0.40–3.54)	0.750
Hausa	2.57 (1.07–6.20)	0.036**	1.0 (0.74–3.82)	0.824
Ga/Dangme	2.3 (0.69–7.86)	0.170	0.52 (0.07–3.93)	0.526
Others	4.91 (0.79–30.64)	0.089*	0.71 (0.09–5.49)	0.739
Educational level				
No formal education	1		1	
Basic	0.50 (0.03–8.31)	0.629	1.81 (0.91–7.63)	0.987
Secondary	11.05(1.39–87.52)	0.023**	1.4 (0.09–20.83)	0.805
Tertiary	16.89 (7.35–40.17)	< 0.001***	17.76 (1.32–23.52)	0.030**
Occupational status				
Employed/Self-Employed	1		1	
Unemployed	0.66 (0.29–1.52)	0.328	1.69 (0.45–6.42)	0.436
Retired	0.55 (0.22–1.38)	0.203	0.55 (0.08–3.84)	0.551
Student	3.36 (1.43–7.93)	0.006***	7.76 (1.10-54.59)	0.040**
Ownership of smartphone				
Yes	1		1	
No	0.07 (0.02–0.20)	< 0.001***	0.26 (0.05–1.51)	0.134

*** *p* < 0.01, ** *p* < 0.05, * *p* < 0.1

## Discussion

The study determined the level of awareness and utilisation of mHealth applications among patients who use the Ho Teaching Hospital. This study on the awareness of mHealth apps found that more than half of respondents were unaware of the existence of these apps. This observation contrasts with a study by Peprah and colleagues on knowledge, attitudes, and use of mHealth technology in Ghana, which reported that 71% of respondents knew how to use mobile phones for healthcare [28]. Similarly, a study by Sam and team reported a 78% awareness rate among their respondents [29]. These differences may be attributed to variations in educational backgrounds, smartphone ownership, and the age of the samples in the various studies. Notably, both studies had a higher percentage of student respondents between 19 and 25 years old. However, the findings of the current study align with previous studies, which observed that despite the growing number of mHealth apps, awareness and usability of these apps remained relatively low [9, 30]. The results demonstrated that respondents’ awareness of mHealth applications was significantly associated with age, sex, education level, occupation, and smartphone ownership. This aligns with evidence from other settings. A study in Australia reported that demographic factors, such as gender, age, and education was strongly associated with awareness and adoption of mHealth services [31]. According to a study conducted in Bangladesh, men were more likely than women to be aware of mobile phone-based healthcare services, with women exhibiting lower levels of knowledge about available mHealth options [32]. This implies that demographic and socioeconomic factors such as age, sex, education, occupation, and smartphone ownership play a critical role in shaping awareness of mHealth applications.

Further, this study found that most respondents relied on social media, friends/family, and advertisements as their source of information on these apps. A few of them, however, learned about these apps through health workers/centres. The low reliance on formal sources for health-related information has been documented in previous studies. The low level of digital literacy, resulting from lack of prioritising or inculcating information and digital literacy contents in educational systems could be attributed to this occurrence [33]. It could also be that the informal sources are effective channels for raising awareness and should be promoted accordingly. Regarding the apps that were popular among the respondents, it came to light that while fitness apps were prevalent, medical education apps were less known. Similar to the findings of this study, previously conducted studies on how health consumers use apps for health monitoring, found that most respondents used the fitness apps compared to other mHealth apps [17, 34]. In the past decade, health consumers have shown greater interest in their physical functioning, supported by advances in health promotion practices [35], which might explain the surge in the knowledge of fitness apps among the study respondents.

This study further found that almost half of the respondents had positive attitudes and perceptions toward mHealth apps, with most believing the apps were effective and helped them efficiently manage their healthcare needs. Previously, Agbenyo and colleagues conducted a study on how mHealth apps are used to access reproductive healthcare in Tamale, Ghana, and reported similar findings [36]. The fact that these apps engage patients in managing their own health and further empower them with health knowledge has been attributed to their positive perception of these apps [37]. Even though the study indicated that most respondents had positive attitudes toward these apps, a significant number had low trust in the information they provided. Although, previous studies, including those by Sam and team, have discovered that only a few people perceive mHealth apps as reliable [29], reasons included the fact that the “proliferation of mobile health apps has largely been without oversight or regulation, with some arguing that the quality of these apps is highly variable”, could be the reason for the low trust in these apps as recorded in this study [38]. The study showed that sex, religion, education, and smartphone ownership were associated with people’s perceptions of mHealth applications. Similarly, a cross-sectional survey conducted in Israel identified demographic factors, such as gender, age, education, marital status, religious affiliation, and perceived health status, as significant determinants of Health Maintenance Organisation–mobile Health (HMO-mHealth) app adoption. Women preferred in-person consultations for personal medical diagnosis and treatment [39]. This will suffice as the reason why only a few of the respondents indicated that they are comfortable sharing their contact or personal details on mHealth apps, and why most would refrain from using apps that are not endorsed by an established health authority. This underscores the importance of rigorous checks and approval processes for these apps.

Regarding actual use of these apps, this study found that less than half of the study respondents had ever used them, with most of these respondents using them a few times a month. This aligns with previous studies that have confirmed that the use and propagation of mHealth by intended users is still insignificant and slow, and that even though most people display a positive attitude toward mHealth, only a few respondents have actually used it [40]. This finding might be attributed to users usually being more reluctant to use health-related innovations than other innovative products [41]. Another key outcome of this study was the significant relationships between mHealth app use and sociodemographic variables, including age, marital status, ethnic background, educational status, occupational status, and smartphone ownership. However, age, educational level, and occupation remained significantly associated with mHealth utilisation. These results align with previous studies, which have also emphasised the influence of demographic factors on the use of mHealth apps among different populations [42]. A study in China reported that older adults faced disadvantages in accessing and using mHealth services (β = − 0.38 and − 0.41), as did individuals with lower levels of education (β = − 0.24 and − 0.27). However, the direct effects of these factors on mHealth use were not statistically significant [41].

Finally, this study also sought to determine why the majority of respondents never use the apps, and a lack of awareness was the prevailing reason. Published research has established a lack of awareness as a major hindrance to the adoption and use of mHealth apps [9, 43]. Hengst and colleagues have attributed the low awareness level and usage to low digital literacy among the citizenry [44]. Integrating digital literacy into the curriculum of public health institutions can enhance awareness of essential health technologies among health professionals, enabling them to guide their communities effectively. Public and medical librarians play a critical role in this context, given their expertise in information management and ability to support the use of health applications.

### Limitations

The cross-sectional design adopted for this study limits the ability to establish causal relationships between examined factors and mHealth awareness, perception, or utilisation. The use of self-reported data introduces the possibility of information bias, including recall and social desirability biases. Although every effort was taken to protect participant anonymity and ensure comfort during interviews, it is impossible to entirely eliminate recall and residual biases. Also, the hospital-based, single-site design may limit the generalisability of the findings beyond the study population. Additionally, sparse data in certain subgroups such as marital status, religion, ethnic background, and educational level led to wide confidence intervals and reduced precision of some regression estimates. Despite these limitations, systematic and standardised sampling procedures were applied to enhance the internal validity and reliability of the study’s findings. Future research should adopt longitudinal or intervention-based designs to better investigate changes in the use of mHealth over time as well as to assess the effectiveness of strategic approaches targeting improvement in awareness, perception, and utilisation of mHealth applications.

## Conclusion

This study found that, despite the increasing availability of mHealth tools, awareness and utilisation remain low. Although respondents generally held positive attitudes toward mHealth applications, concerns about trust and reliability were evident. These results highlight the need for policies that strengthen digital health literacy, promote public awareness through formal health systems, and support quality assurance mechanisms for mHealth applications. Policy efforts should prioritise integrating mHealth education into routine healthcare delivery and national digital health strategies to improve equitable access and informed use. While demographic differences in awareness and utilisation were observed, broader multi-site research is needed to confirm these patterns before highly targeted interventions are implemented. Strengthening regulatory oversight and partnerships between healthcare providers and technology stakeholders may help improve confidence in mHealth tools and support their effective adoption in Ghana.

## Supplementary Information

Supplementary Material 1.

## Data Availability

The anonymised data may be made available by the corresponding author upon reasonable request.

## Abbreviations

MHealth: Mobile health
OPD: Outpatient Department
SMS: Short Message Service
SSA: Sub-Saharan Africa
GSMA: Global System for Mobile Communications Association
ICT /: ICTs Information and Communication Technology / Technologies
VIF: Variance Inflation Factor
