# Supplementary material for: Health consumers’ use of mHealth applications in Ghana: a cross-sectional study
Source: BMC Public Health. 2026 Mar 13;26:1295. doi: 10.1186/s12889-026-26996-z (PMC13097972; doi:10.1186/s12889-026-26996-z)
Supplement: Supplementary file 1 — Supplementary Material 1. [file 12889_2026_26996_MOESM1_ESM.docx]

**STRUCTURED INSTRUMENT**

**TOPIC: Health consumers’ use of mHealth applications in Ghana: A cross-sectional study**

**SECTION A: Demographic Characteristics**

| S/N | Question | | Responds (Thick as required) | |
| --- | --- | --- | --- | --- |
| A1 | What is your Age (years)? | | ……………………………… | |
| A2 | What is your Sex? | | Male [ ]  Female [ ] | |
| A3 | What is your Marital status? | | Single [ ]  Married [ ]  Separated/Divorced [ ]  Widowed [ ] | |
| A4 | What is your Religion? | | Christian [ ]  Muslim [ ]  African Traditional Religion [ ]  Others [ ] | |
| A5 | What is your Ethnicity? | | Ewe [ ]  Akan [ ]  Hausa [ ]  Ga/Dangme [ ]  Fante [ ]  Others (Specify) ………. | |
| A6 | What is your highest level of education | | No formal education [ ]  Basic [ ]  Secondary [ ]  Tertiary [ ] | |
| A7 | What is your Occupation? | | Employed/Self-employed [ ]  Unemployed [ ]  Retired [ ]  Student [ ] | |
| A8 | Do you own a Smartphone? | | Yes [ ]  No [ ] | |
| **Section B: Awareness of Mobile Health Applications (mHealth Apps)** | | | | |
| B1 | | Are you aware of what we call Mobile Health (mHealth) Applications? | | Yes [ ]  No [ ] |
| B2 | | If yes to Question B1, where have you heard about mHealth Apps? | | Social media [ ]  Advertisements [ ]  Family or friends [ ]  Health workers or health center [ ]  Others (Specify) ………….. |
| B3 | | Which mHealth Apps do you know about? | | Fitness apps [ ]  Vital signs tracker apps (e.g. Blood Pressure, Heart rate, Blood sugar, Weight and Height etc.) [ ]  My calendar (period tracker) [ ] Medical education apps [ ]  Diet and nutrition apps [ ] Others (Specify) ………….. |
| **Perceptions About mHealth Apps and Its Effectiveness** | | | | |
| C1 | | Using mHealth Apps helps manage healthcare needs more efficiently? | | Agree [ ]  Disagree [ ]  Neutral [ ] |
| C2 | | Using mHealth Apps increases my ability to manage my own health? | | Agree [ ]  Disagree [ ]  Neutral [ ] |
| C3 | | Using mHealth Apps is cost effective (not expensive)? | | Agree [ ]  Disagree [ ]  Neutral [ ] |
| C4 | | Using mHealth Apps saves lots of time? | | Agree [ ]  Disagree [ ]  Neutral [ ] |
| C5 | | Using mHealth Apps is safe and secure? | | Agree [ ]  Disagree [ ]  Neutral [ ] |
| C6 | | Information obtained from mHealth Apps are reliable and accurate? | | Agree [ ]  Disagree [ ]  Neutral [ ] |
| C7 | | I am comfortable sharing my personal or contact details on mHealth apps? | | Agree [ ]  Disagree [ ]  Neutral [ ] |
| C8 | | I am willing to try or use mHealth Apps? | | Agree [ ]  Disagree [ ]  Neutral [ ] |
| C9 | | I would not use a mHealth app that is not supported by a recognized health authority, such as the Ghana Health Service (GHS)? | | Agree [ ]  Disagree [ ]  Neutral [ ] |
| **Utilization of mHealth Apps** | | | | |
| D1 | | Have you ever used any mHealth App? | | Yes [ ]  No [ ] |
| **If Yes to Question D1, answer Question D2 to D5** | | | | |
| D2 | | Which mHealth Apps have you ever used? | | Fitness apps [ ]  Vital signs tracker apps (e.g. Blood Pressure, Heart rate, Blood sugar, Weight and Height etc.) [ ]  My calendar (period tracker) [ ] Medical education apps [ ]  Diet and nutrition apps [ ] Others (Specify) |
| D3 | | How often do you use the mHealth Apps? | | Daily [ ]  Few times in a week [ ]  Few times in a month [ ]  I barely use them [ ] |
| D4 | | Why do you use mHealth Apps? | | Helps me track my health [ ]  It motivates me [ ]  It provides useful reminders [ ]  It is fun or simple to use [ ]  Others (Specify) ………….. |
| D5 | | Are you willing to recommend mHealth Apps to your friends and family to use? | | Yes [ ]  No [ ] |
| **Answer Question D6 if your response to Question D1 was ‘No’** | | | | |
| D6 | | Why do you not use mHealth Apps? | | I am not aware of them [ ]  Time constraints [ ]  They are not effective [ ]  Information shared with them are not safe and secured [ ]  Others (Specify) ………….. |
